# Effect of short schemes on body composition measurements using Air-Displacement Plethysmography

**DOI:** 10.1186/1476-5918-4-8

**Published:** 2005-07-26

**Authors:** Holly R Hull, David A Fields

**Affiliations:** 1 Department of Health and Exercise Sciences, University of Oklahoma, Norman, OK, USA; 2 Department of Pediatrics, University of Oklahoma Health Science Center, Oklahoma City, OK, USA

## Abstract

**Background:**

Air-displacement plethysmography (ADP) is becoming a popular method to assess body composition. Several studies have shown certain types of clothing can affect measurements of body density, however no study has specifically investigated the effect of cotton gym shorts and spandex bicycle shorts on body density.

**Methods:**

Thirty-seven males (23.0 ± 3.2 yr., 177.3 ± 5.4 cm., 74.8 ± 7.5 kg.) and thirty-eight females (23.7 ± 5.3 yr., 163.6 ± 8.4 cm., 57.1 ± 7.0 kg.) had their body density measured by ADP in three clothing schemes: 1) a tight fitting Speedo^® ^swim suit (criterion measure), 2) cotton gym shorts, and 3) spandex bicycle shorts. The clothing was provided by the University of Oklahoma Body Composition Laboratory and the testing schemes were performed in random order.

**Results:**

The regression of body density by the criterion measure against body density while wearing cotton gym shorts for the entire group (y = 0.001 + 0.991x, SEE = 0.003 g/cm^3^) and the females (y = 0.059 + 0.934x, SEE = 0.003 g/cm^3^) did not significantly deviate from the line of identity. However in males the regression significantly deviated from the line of identity (y = 0.052 + 0.944x, SEE = 0.002 g/cm^3^). Body density by the criterion measure and body density while wearing spandex bicycle shorts did not significantly differ from the line of identity for the entire group (y = -0.018 + 1.013x SEE = 0.003 g/cm^3^), in males (y = -0.002 + 1.001x, SEE = 0.003 g/cm^3^), or females (y = 0.073 + 0.925x, SEE = 0.003 g/cm^3^). Residual plot analysis revealed no group or gender bias in either the cotton gym shorts or in the spandex bicycle shorts.

**Conclusion:**

It would appear bicycle spandex shorts are an acceptable alternative to a Speedo^® ^like swim suit, however we advise that subjects adhere to the strict clothing protocol that is recommended by the manufacturer.

## Background

Air-displacement plethysmography (ADP) is gaining wide approval and use in the assessment of body composition in not only research laboratories but athletic facilities as well. ADP is commonly used in place of more established methods (e.g., hydrostatic weighing and dual energy X-ray absorptiometry) in part because of a short assessment time but also due to high subject compliance [1]. Body density measurements are quickly and easily assessed through the measurement of body volume by use of air-displacement plethysmography [2].

The operating principles, physical design and testing procedures for ADP have been described elsewhere [1,2]. Related specifically to testing procedures for ADP, one important issue warranting further consideration is clothing worn during testing. Body density is estimated through the measurement of body volume. ADP measures body volume through the application of Boyle's law which states at constant (isothermal) temperature, pressure and volume are inversely related. Therefore, air compressed under isothermal conditions will decrease volume in proportion to increasing pressure. However, under adiabatic conditions (occurring without loss or gain of heat), the temperature of the air does not remain constant as volume changes and the molecules gain or lose kinetic energy [2]

When a subject enters the enclosed testing chamber for a body volume measurement, the conditions are adiabatic. Adiabatic conditions are created by heat loss at the skin and normal ventilation by the subject causing changes in air temperature and pressure inside the chamber without a concurrent change in volume. Air next to the skin and air trapped in hair and the fabric of clothing worn during testing are at isothermal conditions [2]. These isothermal conditions must be taken into account or body fat can be underestimated by as much as 6% [3,4]. Steps are taken to minimize the effect of hair and clothing and include recommendations made by the manufacturer to have the subject wear a minimal amount of clothing while being tested. This includes a tight fitting swim cap with all hair tucked inside the cap and a tight fitting one-piece swim suit for females and a tight fitting brief like swimsuit for males.

If steps are not taken to control for isothermal air trapped in the fabric of clothing, invalid body density measurements are obtained. Two studies have investigated how trapped air in clothing affects body density measurements [3,4]. Specifically, both studies compared wearing a one-piece swimsuit to wearing a hospital gown and the resulting affects on body density measurements. They found a significant overestimation of body density resulting in an approximate 6% underestimation of percent fat (%fat).

With increased use of ADP in research laboratories and athletic facilities, it has been reported subjects were allowed to be tested in common athletic shorts or spandex bicycle shorts [5,6]. At this time, we are unaware of any studies that have specifically investigated the effect of different clothing schemes (i.e. cotton gym shorts and spandex bicycle shorts) on measurements of body density. Therefore, the purpose of this study was to examine the effect of different types of short schemes on measurements of body density and %fat.

## Methods

### Subjects

Seventy-five adult subjects (37 males and 38 females) ranging in age from 18–40 years old gave their informed consent to participate in the study. The study design and testing procedure was explained prior to obtaining informed consent from each participant and approval for the use of human subjects was obtained from the Institutional Review Board from the University of Oklahoma at Norman.

### Protocol

Subjects reported to the body composition laboratory for testing after a four hour fast. Height was measured using a wall mounted stadiometer (Accu-Hite Wall Stadiometer, Seca Corp., Hanover, MD) and body weight was measured to the nearest 0.01 kg using the BOD POD^® ^system electronic scale, described previously [2]. All testing was completed in random order by use of a randomization table with total body density measured in the following three clothing schemes: 1) a tight fitting Speedo^® ^swimsuit (criterion measure), 2) cotton gym shorts, and 3) spandex bicycle shorts. Each of the three short schemes was provided by the body composition laboratory.

#### BOD POD instrumentation

Whole body air-displacement was completed with the BOD POD^® ^version 1.69 (Body Composition System; Life Measurement, Incorporated, Concord, CA) as previously described [1]. Calculation of %fat is derived from assessment of body volume based on the following equation:

Body Volume (l) = Body Volume_raw _(1) - Surface Area Artifact (l) + 40% Thoracic Gas Volume (l)

Once body volume was determined, body density was then calculated by dividing mass of the subject by body volume. All subjects were tested in a standardized Speedo^® ^swimsuit and swim cap that was provided by the body composition laboratory. Thoracic gas volume was measured in all subjects and for all short schemes with %fat being determined by the Siri equation [7]. The day-to-day coefficient of variation for the BOD POD in our laboratory is 1%.

### Data analysis

Group and gender mean estimates of %fat wearing either a Speedo^® ^(considered the criterion), cotton gym shorts, and spandex bicycle shorts was compared using paired t-tests. Accuracy and bias was examined by comparing body density while wearing a Speedo^® ^to body density while wearing cotton gym shorts and spandex bicycle shorts.

Regression analysis was used to determine the agreement between the criterion measure and the two short schemes (cotton gym shorts and spandex bicycle shorts) for body density. Measurements were considered accurate if the regression between the criterion measure and the short scheme had a slope not significantly different from one and an intercept not significantly different from zero. Residual plot analysis examined potential bias between the criterion measure and the short schemes where a non-significant correlation suggests no bias in the technique across the range of fatness [8]. This analysis involves an assessment of correlation or the measure of strength of the relation between the mean of the criterion measure correlated to the difference between a short scheme minus the criterion. This provides insight into how much the short scheme differs and relates to the criterion. Statistical significance was set at an alpha value of *P *≤ 0.05. The Statistical Package for the Social Sciences (SPSS, Version 10.0 for Windows) was used for all analyses.

## Results

The physical characteristics and group mean measurements of body density by the different short schemes for the entire group and by gender are presented in Table 1.

**Table 1:** Descriptive characteristics for subjects and body composition variables.

**Variable**	**Males (n = 37)**	**Females (n = 38)**	**Group (n = 75)**
Age (yr)	23.0 ± 3.2	23.7 ± 5.3	23.3 ± 4.4
Height (cm)	177.3 ± 5.4†	163.6 ± 8.4	170.3 ± 9.8
Body Weight (kg)	74.8 ± 7.5†	57.1 ± 7.0	65.8 ± 11.5
BMI (kg/m^2^)	23.8 ± 2.3	21.4 ± 2.7	22.6 ± 2.3
Criterion (% fat)	16.9 ± 6.4	24.7 ± 5.1	20.8 ± 6.9
Criterion (g/cm^3^)	1.060 ± 0.014	1.042 ± 0.011	1.052 ± 0.016
Cotton Gym Short (g/cm^3^)	1.067 ± 0.015*	1.052 ± 0.016*	1.059 ± 0.015*
Spandex Bicycle Short (g/cm^3^)	1.062 ± 0.014*	1.047 ± 0.011*	1.055 ± 0.015*

*Significantly different from Criterion (*P *≤ 0.05)

† Significant gender difference (*P *≤ 0.05)

The first series of regression analysis examined the accuracy of the different clothing schemes by the regression of body density by the criterion (i.e. wearing a Speedo^®^) against the body density by the two clothing schemes for the entire group and by gender.

**Figure 1:**
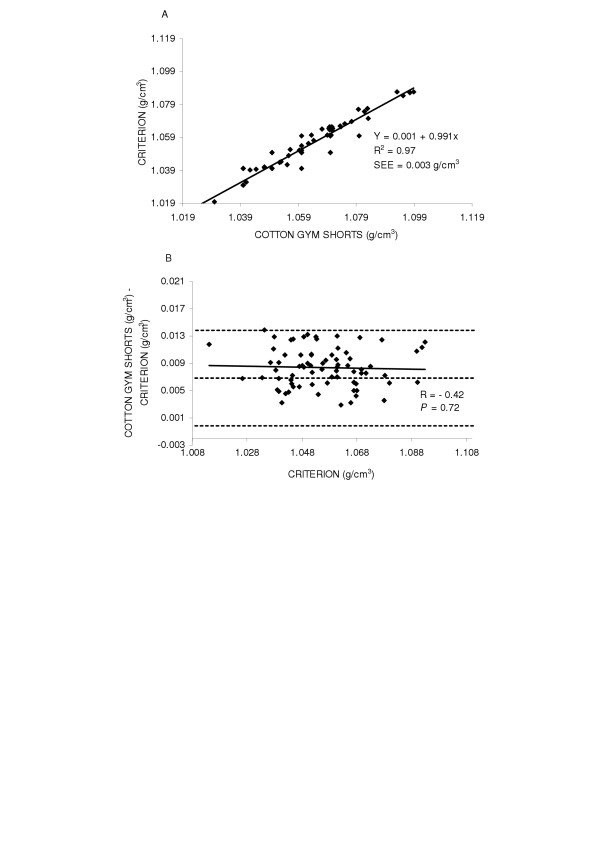
Panel A is the regression of body density (g/cm^3^) by the criterion against body density by cotton gym shorts for the group. Panel B is the residual plot for the group where the middle dashed line represents the mean difference between body density by the criterion - body density by cotton gym shorts. The upper and lower dashed lines represents ±2 SD from the mean. No bias between the techniques was observed as indicated by a non-significant *P *value.

**Figure 2:**
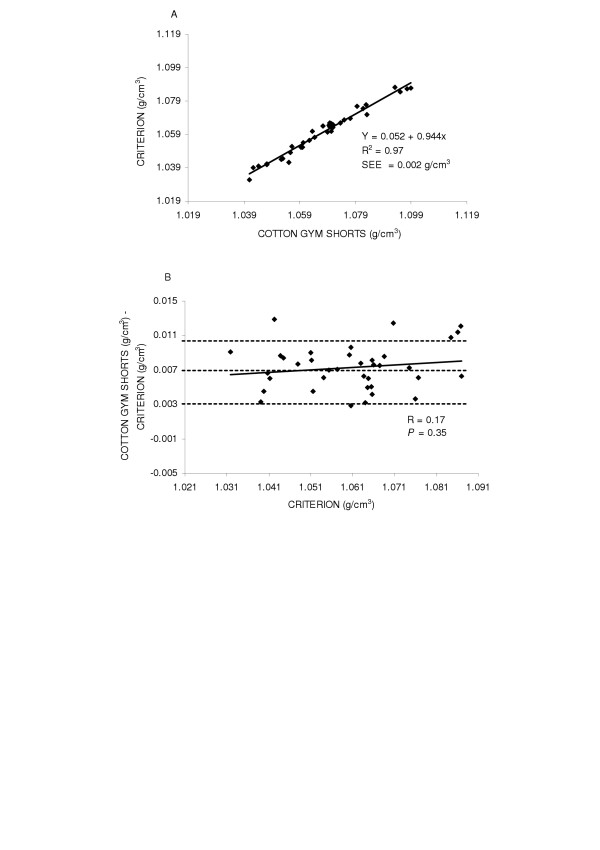
Panel A is the regression of body density (g/cm^3^) by the criterion against body density by cotton gym shorts in males. Panel B is the residual plot in males where the middle dashed line represents the mean difference between body density by the criterion - body density by cotton gym shorts. The upper and lower dashed lines represents ±2 SD from the mean. No bias between the techniques was observed as indicated by a non-significant *P *value.

**Figure 3:**
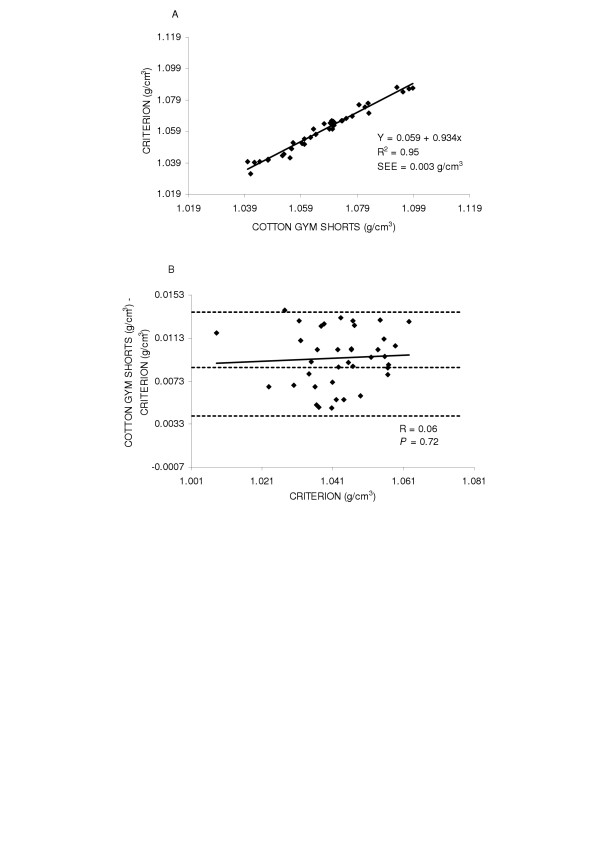
Panel A is the regression of body density (g/cm^3^) by the criterion against body density by cotton gym shorts in females. Panel B is the residual plot in females where the middle dashed line represents the mean difference between body density by the criterion - body density by cotton gym shorts. The upper and lower dashed lines represents ±2 SD from the mean. No bias between the techniques was observed as indicated by a non-significant *P *value.

First, the accuracy of body density was examined by the regression of body density by the criterion against the body density while wearing cotton gym shorts for the entire group (y = 0.001 + 0.991x SEE = 0.003 g/cm^3^, R^2 ^= 0.97) and found the regression did not deviate from the line of identity (Figure 1 top panel (A)). Next potential gender differences were explored. The regression between body density by the criterion method and body density while wearing cotton gym shorts are shown in Figures 2 and 3 top panels (A) for males and females, respectively. In females (y = 0.059 + 0.934x, SEE = 0.003 g/cm^3^, R^2 ^= 0.95) the regression did not significantly deviate from the line of identity. However in males the regression significantly deviated from the line of identity (y = 0.052 + 0.944x, SEE = 0.002 g/cm^3^, R^2 ^= 0.97).

**Figure 4:**
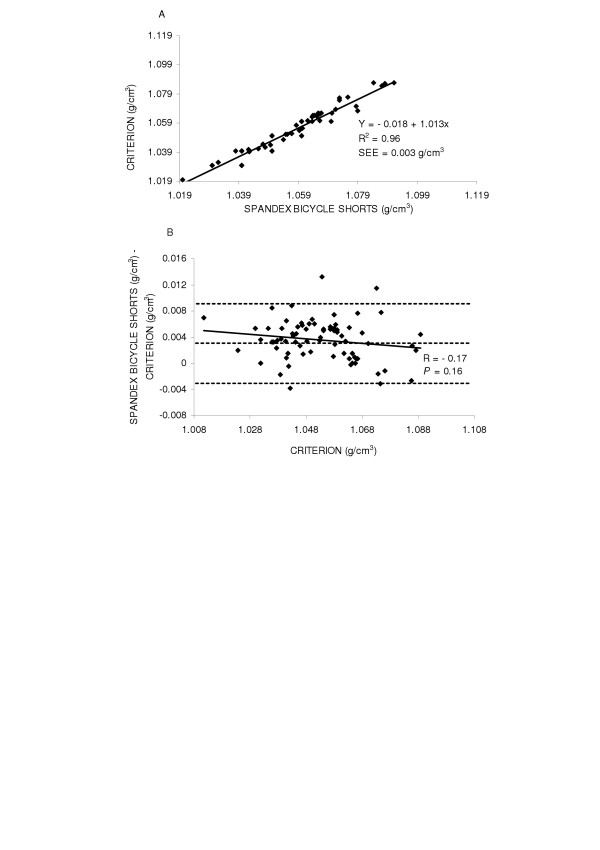
Panel A is the regression of body density (g/cm^3^) by the criterion against body density by spandex bicycle shorts for the group. Panel B is the residual plot for the group where the middle dashed line represents the mean difference between body density by the criterion - body density by spandex bicycle shorts. The upper and lower dashed lines represents ±2 SD from the mean. No bias between the techniques was observed as indicated by a non-significant *P *value.

**Figure 5:**
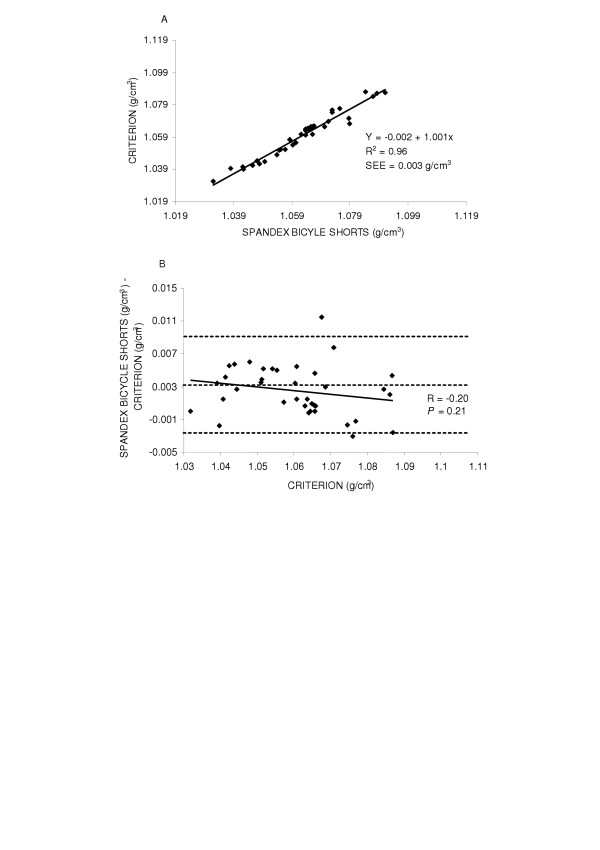
Panel A is the regression of body density (g/cm^3^) by the criterion against body density by spandex bicycle shorts in males. Panel B is the residual plot in males where the middle dashed line represents the mean difference between body density by the criterion - body density by spandex bicycle shorts. The upper and lower dashed lines represents ±2 SD from the mean. No bias between the techniques was observed as indicated by a non-significant *P *value.

**Figure 6:**
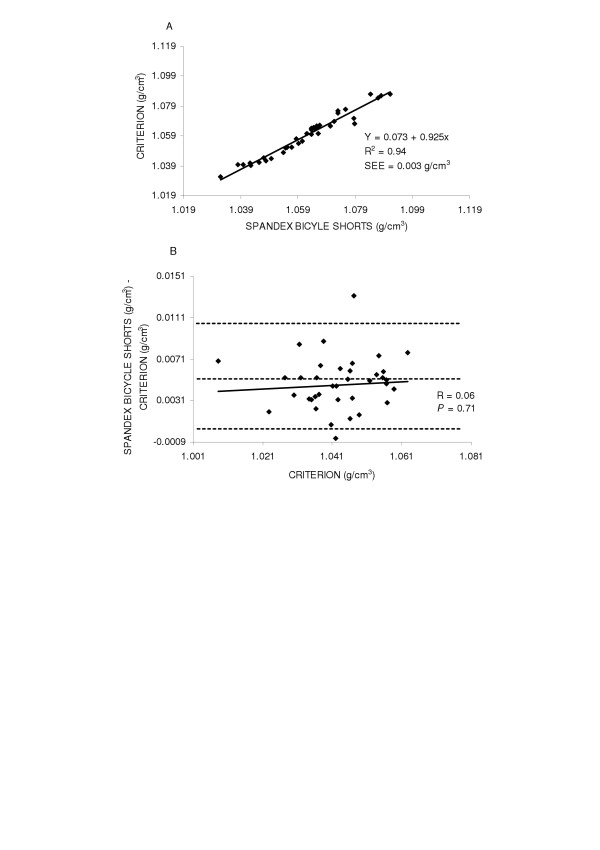
Panel A is the regression of body density (g/cm^3^) by the criterion against body density by spandex bicycle shorts in females. Panel B is the residual plot in females where the middle dashed line represents the mean difference between body density by the criterion - body density by spandex bicycle shorts. The upper and lower dashed lines represents ±2 SD from the mean. No bias between the techniques was observed as indicated by a non-significant *P *value.

The accuracy of the spandex bicycle shorts were examined by the regression of body density by the criterion method against body density while wearing spandex bicycle shorts for the entire group and by gender. The regression comparing body density by the criterion method and body density while wearing spandex bicycle shorts did not significantly deviate from the line of identity for the entire group (y = -0.018 + 1.031x, SEE = 0.003 g/cm^3^, R^2 ^= 0.96), in males (y = -0.002 + 1.001x, SEE = 0.003 g/cm^3^, R^2 ^= 0.96) or in females (y = 0.073 + 0.925x, SEE = 0.003 g/cm^3^, R^2 ^= 0.94) Figures 4, 5 and 6 top panels (A) respectively.

Residual plot analysis was performed for each clothing scheme to determine potential bias across the range of body fatness. First, a group comparison while wearing cotton gym shorts and spandex bicycle shorts was completed and are shown in Figures 1 and 4 bottom panel (B), respectively. No bias was observed while wearing cotton gym shorts (R = -0.42, *P *= 0.72) or while wearing spandex bicycle shorts (R = -0.17, *P *= 0.16). The next series of plots for cotton gym shorts are shown in Figures 2 and 3 bottom panels (B) for males and females respectively and the plots for spandex bicycle shorts are shown in Figures 5 and 6 bottom panels (B) for males and females respectively. No bias was observed in either males (R = 0.17, *P *= 0.35) or females (R = 0.06, *P *= 0.72) while wearing cotton gym shorts or while wearing spandex bicycle shorts in either the males (R = -0.20, *P *= 0.21) or females (R = 0.06, *P *= 0.71) as indicated by non-significant p-values.

**Figure 7:**
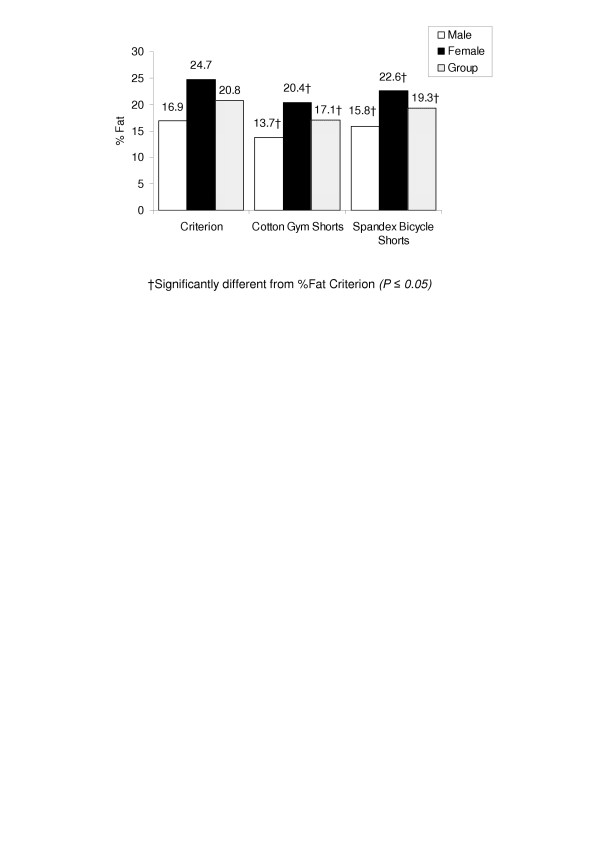
The bars represent the mean %fat values for each technique for males, females and the entire group.

Significant differences (*P *< 0.05) were observed when comparing %fat, between the criterion and both shorts schemes for the entire group, and in both males and respectively (Figure 7).

## Discussion

This study found a significant under-estimation between typical cotton gym shorts and the criterion clothing scheme, however this difference did not exist for the spandex shorts. The manufacturer recommends wearing a tight fitting swimsuit during testing due to differing responses of gas under adiabatic and isothermal conditions. Two studies have examined the effect of clothing on body density, however in each study the clothing examined was a hospital gown [3,4]. This represented a gross distortion of the capability of the BOD POD^® ^to resolve isothermal air and violates the manufacturer recommendation of wear a tight fitting swimsuit, though it does represent a clinical application. Fields et al., and Vescovi et al., studied the effect of wearing a hospital gown in college aged females and found a significant overestimation of body density (~0.016 g/cm^3^) which represents an approximate 6%fat underestimation when compared to normal testing conditions (i.e. tight fitting swim suit) [3,4]. Although cotton gym shorts do not contain the same amount of fabric as a hospital gown, regression results from our study found a significant difference between the two methods, resulting in an overestimation of body density for males (0.007 g/cm^3^, ~3%fat males).

In theory, spandex bicycle shorts have more fabric than a swimsuit but due to the tight fitting nature, they have less capability to trap air and therefore less ability to affect body density. However, regression analysis from our study comparing body density while wearing spandex bicycle shorts found no significant difference for either gender (0.002 g/cm^3 ^males and 0.005 g/cm^3 ^females) or by group comparisons. Of note, the manufacturers indicate testing while wearing spandex bicycle shorts is an acceptable testing condition.

Air trapped in hair and clothing is isothermal and will be more compressible during body volume measurements. This results in a decrease in body volume and therefore an overestimation of body density and an underestimation of %fat [2]. The effect of isothermal air on estimates of %fat was investigated in a study by Higgins et al., where males were tested to examine the effect of body hair on ADP measurements [9]. The subjects were tested in the following conditions and then compared to a criterion method (beard shaved and swim cap worn): 1) facial hair and swim cap, 2) facial hair and no swim cap, and 3) no facial hair and no swim cap. Results indicated the presence of a beard or scalp hair (no swim cap worn) resulted in a significant underestimation of %fat (1.4%fat). This study demonstrates that even small changes in air conditions can have a relative modest impact on %fat estimates.

ADP is growing in popularity and is increasingly utilized in research laboratories and athletic facilities across the country. In situations where a high volume of testing occurs, the subjects clothing attire can present time and financial constraints. To outfit a diverse population of not only of males and females and young and old, but also varying sizes, a wide range of bathing suit sizes and styles must be available (a brief like suit for males and a one piece suit for females). In most athletic facilities and clinical settings, this is costly and unrealistic. An additional issue that arises is that each subject would need a clean swimsuit further adding to the amount of testing attire required.

From a practical standpoint, the findings from our study are of important significance because often time subjects and athletes are tested in cotton gym shorts or spandex bicycle shorts out of convenience and for improved subject compliance. Often times, athletes are tested in standard issued athletic clothing which the athlete receives at the beginning of there athletic season. Due to comfort and ease, athletes are tested in this athletic wear which usually consists of either spandex bicycle shorts or cotton athletic gym shorts. We do understand that for either fit athletes or community participants it may be more comfortable and less embarrassing to have body composition assessed while wearing shorts; however we would urge researchers and clinicians to avoid this convenience and adhere to a strict testing protocol (i.e., a Speedo^® ^like swim suit).

A specific example of assessing body composition in an athletic population in clothing that is convenient rather than recommended is a study by Collins et al., in a population of collegiate football players [6]. The study sought to compare body composition measurements from ADP to measurements from hydrostatic weighing and dual energy x-ray absorptiometry in 20 Division 1A football players. Study protocol describes testing occurred while subjects were wearing Lycra shorts and not the recommended brief like swim suit [6]. Collins et al., reported the mean body density ADP was significantly greater than body density hydrostatic weighing (*P *< 0.05) and the slope from the regression analysis was significantly different than 1 (*P *< 0.05) [6]. Comparing ADP to dual energy x-ray absorptiometry found %fat ADP (10.9 ± 1.0%) was significantly less than %fat DXA (12.9 ± 1.2%). In the discussion, Collins et al., noted that the testing attire used deviated from the manufacturers recommended protocol and may have contributed to the observed differences. A second study by Vescovi et al., illustrates testing in the general population when the testing clothing used was based upon convenience or comfort [10]. Study protocol stated a minimal amount of clothing was worn during testing that included spandex shorts or a swimsuit. This study compared ADP to hydrostatic weighing in a group of males and females ranging in age from 18–52 years. No significant difference between ADP and hydrostatic weighing was observed. Further analysis classified subjects lean, average and overweight. In the lean subset of subjects, a significant difference was found between ADP and hydrostatic weighing (*P *< 0.001). It was not indicated within each subset which subjects wore a swimsuit and which subjects wore spandex shorts. Since no differentiation was made of what testing attire each subject wore while being tested, it is unknown if this had an affect on the results. It may be possible the differences found between ADP and hydrostatic weighing in a subset of lean subjects were related to amount of clothing worn instead to actual differences between techniques.

In conclusion, our results indicate that testing in males while wearing cotton gym shorts resulted in an approximate 3% underestimation of body fat while testing wearing spandex bicycle shorts appears to be an acceptable alternative. However, we recommend that all subjects wear a tight fitting swimsuit while having their body composition assessed by ADP to eliminate any error that could occur.

**Table 2:** Summary of regression of %fat estimates by the criterion measure vs. the other short schemes.

**Group**	**Intercept**	**Slope**	**R^2^**	**SEE**
Cotton Gym Shorts	3.52*	1.01	0.97	1.29
Spandex Bicycle	1.24*	1.02	0.96	1.40
**Males**				
Cotton Gym Shorts	3.73*	0.96	0.97	1.09
Spandex Bicycle	1.06	1.00	0.96	1.35
**Females**				
Cotton Gym Shorts	5.20*	0.95	0.95	1.21
Spandex Bicycle	3.53*	0.94	0.94	1.28

* Significantly different from 0

## References

[B1] McCrory MA, Gomez TD, Bernauer EM, Mole PA (1995). Evaluation of a new air displacement plethysmography for measuring human body composition. Med Sci Sports Exerc.

[B2] Dempster P, Aitkens S (1995). A new air displacement method for the determination of human body composition. Med Sci Sports Exerc.

[B3] Fields DA, Hunter GR, Goran MI (2000). Validation of the BOD POD with hydrostatic weighing: influence of body clothing. Int J Obes Relat Metab Disord.

[B4] Vescovi JD, Zimmerman SL, Miller WC, Fernhall B (2002). Effects of clothing on accuracy and reliability of air displacement plethysmography. Med Sci Sports Exerc.

[B5] Wagner DR, Heyward VH, Gibson AL (2000). Validation of air displacement plethysmography for assessing body composition. Med Sci Sports Exerc.

[B6] Collins MA, Millard-Stafford ML, Sparling PB, Snow TK, Rosskopf LB, Webb SA, Omer J (1999). Evaluation of the BOD POD for assessing body fat in collegiate football players. Med Sci Sports Exerc.

[B7] Siri WE, Bozek J and Henschel A (1961). Body composition from fluid spaces and density: analysis of methods.. Techniques for Measuring Body Composition.

[B8] Bland JM, Altman DG (1986). Statistical methods for assessing agreement between two methods of clinical measurement. Lancet.

[B9] Higgins PB, Fields DA, Hunter GR, Gower BA (2001). Effect of scalp and facial hair on air displacement plethysmography estimates of percentage of body fat. Obes Res.

[B10] Vescovi JD, Zimmerman SL, Miller WC, Hildebrandt L, Hammer RL, Fernhall B (2001). Evaluation of the BOD POD for estimating percentage body fat in a heterogeneous group of adult humans. Eur J Appl Physiol.

